# Forty Years of the Description of Brown Spider Venom Phospholipases-D

**DOI:** 10.3390/toxins12030164

**Published:** 2020-03-06

**Authors:** Luiza Helena Gremski, Hanna Câmara da Justa, Thaís Pereira da Silva, Nayanne Louise Costacurta Polli, Bruno César Antunes, João Carlos Minozzo, Ana Carolina Martins Wille, Andrea Senff-Ribeiro, Raghuvir Krishnaswamy Arni, Silvio Sanches Veiga

**Affiliations:** 1 Departamento de Biologia Celular, Universidade Federal do Paraná (UFPR), Curitiba 81531-980, PR, Brazil; luizagremski@ufpr.br (L.H.G.); hannajusta@gmail.com (H.C.d.J.); thaiscwb@yahoo.com.br (T.P.d.S.); nayannepolli@gmail.com (N.L.C.P.); brunocesarantunes@hotmail.com (B.C.A.); senffribeiro@gmail.com (A.S.-R.); 2 Centro de Produção e Pesquisa de Imunobiológicos (CPPI), Piraquara 83302-200, PR, Brazil; jminozzo@yahoo.com.br; 3 Departamento de Biologia Estrutural, Molecular e Genética, Universidade Estadual de Ponta Grossa, Ponta Grossa 84030-900, PR, Brazil; anacarolina.wille@yahoo.com.br; 4 Centro Multiusuário de Inovação Biomolecular, Departamento de Física, Universidade Estadual Paulista (UNESP), São José do Rio Preto 15054-000, SP, Brazil; raghuvir.arni@unesp.br

**Keywords:** brown spider, venom, phospholipases-D, biochemical and biological activities

## Abstract

Spiders of the genus *Loxosceles*, popularly known as Brown spiders, are considered a serious public health issue, especially in regions of hot or temperate climates, such as parts of North and South America. Although the venoms of these arachnids are complex in molecular composition, often containing proteins with distinct biochemical characteristics, the literature has primarily described a family of toxins, the Phospholipases-D (PLDs), which are highly conserved in all *Loxosceles* species. PLDs trigger most of the major clinical symptoms of loxoscelism i.e., dermonecrosis, thrombocytopenia, hemolysis, and acute renal failure. The key role played by PLDs in the symptomatology of loxoscelism was first described 40 years ago, when researches purified a hemolytic toxin that cleaved sphingomyelin and generated choline, and was referred to as a Sphingomyelinase-D, which was subsequently changed to Phospholipase-D when it was demonstrated that the enzyme also cleaved other cellular phospholipids. In this review, we present the information gleaned over the last 40 years about PLDs from *Loxosceles* venoms especially with regard to the production and characterization of recombinant isoforms. The history of obtaining these toxins is discussed, as well as their molecular organization and mechanisms of interaction with their substrates. We will address cellular biology aspects of these toxins and how they can be used in the development of drugs to address inflammatory processes and loxoscelism. Present and future aspects of loxoscelism diagnosis will be discussed, as well as their biotechnological applications and actions expected for the future in this field.

## 1. Molecular Characteristics of Brown Spider Venoms

Loxoscelism, the set of clinical manifestations of envenomation by Brown Spiders (*Loxosceles* genus), can be cutaneous and systemic. At the bite site, the cutaneous symptoms are edema, erythema and dermonecrosis with gravitational spreading of lesion (the characteristic hallmark of envenoming). The systemic condition, which is less common, may lead to death and includes hematological changes such as intravascular hemolysis, disseminated intravascular coagulation and thrombocytopenia, as well as acute renal failure [1,2,3,4,5,6]. The volume of venom injected at the time of the bite is low, normally just a few microliters and contains between 60 and 100 micrograms of protein [7]. *Loxosceles* spider venoms are colorless liquids, predominantly composed of proteins and peptides with molecular masses in the 5 to 45 kDa range [2,5]. The toxins encountered in Loxoscelic venoms can be separated into two major groups: (i) insecticidal toxins (Inhibitor Cystine Knot—ICK—peptides), metalloproteases (Astacins) and phospholipases-D (PLDs), and (ii) the toxins expressed in lower quantities such as; hyaluronidases, serine proteases, serine protease inhibitors (serpins), allergenic factors and a translationally controlled tumor protein (TCTP) [5,8]. The total venom composition was determined by transcriptome analyses of the *Loxosceles intermedia* venom gland [8]. The toxin-encoding transcripts quantitative profile of *L. intermedia* venom gland was 56% of Knottins, 23% of Astacins, 20% of PLDs and about 1% of the other toxins [8]. The study of *L. laeta* venom gland transcripts encoding PLDs showed that these toxins correspond to about 16% of all transcripts [9], although 16 clusters annotated as similar to sphingomyelinase D (PLDs) are in fact similar to *Loxosceles* ICK peptides (GenBank accession numbers EY189720, EY188468, EY188410, EY189491, EY189459, EY189456, EY188675, EY189643, EY189620, EY189608, EY188487, EY189575, EY188624, EY188618, EY188594, EY188592) and one similar to other neurotoxins (EY188603) when compared to GenBank sequences using the blastx algorithm (*E* values < 1.00 ×10^−5^). A similar study of transcripts encoding PLDs from *L. similis* venom glands showed that these toxins correspond to about 15% of the total produced transcripts. Transcript analysis revealed 12 main groups and a predominance of s1 and s11 isoforms, which together correspond to about 72% of transcripts for PLDs [10]. The high abundance of these isoforms is not surprising, since *L. similis* PLDs of s1 and s11 groups are highly similar to *L. intermedia* PLDs that comprise 69.7% of *L. intermedia* PLD transcripts. Interestingly, an antimicrobial peptide (1695.75 Da), recently identified in *L. gaucho* venom, presented a remarkable similarity to specific regions of phospholipases D from *Loxosceles* genus. This peptide, referred to as U1-SCRTX-Lg1a, possesses an antibacterial effect on gram-negative bacteria [11]. The authors suggested that U1-SCRTX-Lg1a may have originated by limited proteolysis of a venom PLD. Thus, it is possible that PLD rates evaluated by transcriptomic analyses may be overestimated. 

**Figure 1:**
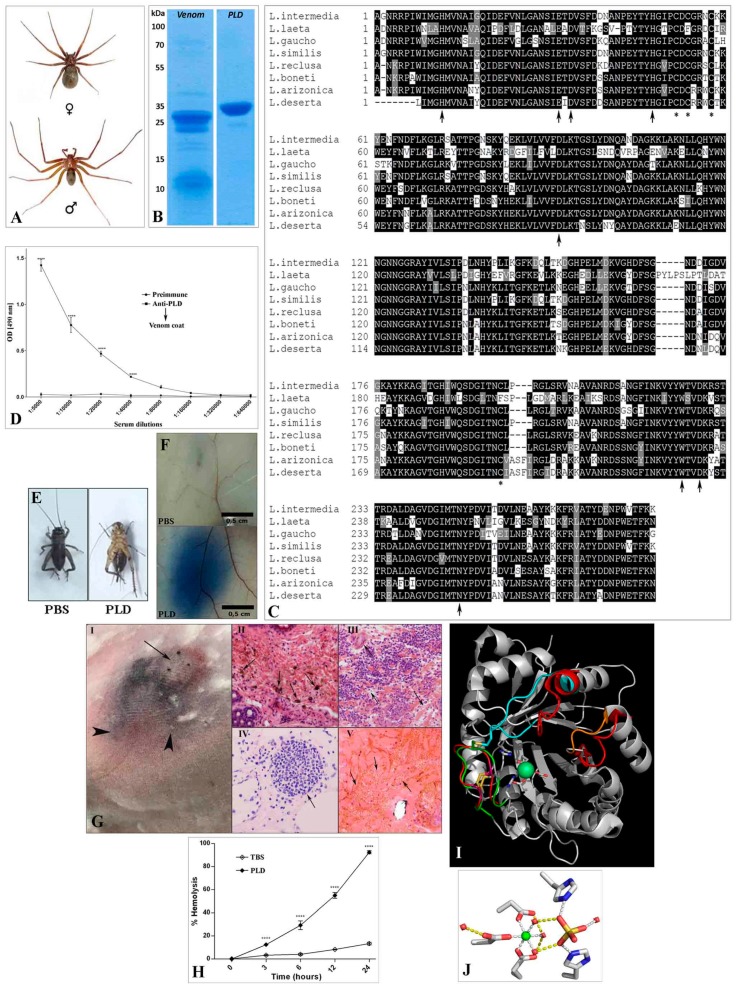
*Loxosceles* phospholipases-D: general aspects and biological activities. (**A**) Sexual dimorphism between female and male adult *L. intermedia* specimens. (**B**) SDS-PAGE under reduced conditions of *L. intermedia* venom (10 µg) and phospholipase-D (PLD) LiRecDT1 (5 µg). (**C**) Multiple sequence alignment of representative phospholipases-D of *L. intermedia* (GenBank accession number ABA62021)*, L. laeta* (AY093599)*, L. gaucho* (JX866729)*, L. similis* (AAX78234)*, L. reclusa* (AY862486)*, L. boneti* (AY559844)*, L. arizonica* (AY699703) and *L. deserta* (C0JAU5). Sequences were aligned using the CLUSTAL X2 program [19]. Amino acid identities are shaded in black. Conservative substitutions are in gray, arrows point to amino acid residues involved in catalysis. The asterisks indicate cysteine residues. (**D**) Reactivity against *L. intermedia* crude venom using different dilutions of anti-LiRecDT1 serum (Anti-PLD) accessed by ELISA. The average ± standard errors are shown, with significance levels ****p ≤ 0.0001 comparing pre-immune with anti-PLD sera. (**E**) Representative images of crickets (n = 5) treated with Phosphate Buffered Saline (PBS) or LiRecDT1 (*L. intermedia* PLD, 4 μg) injected in the second segment of abdomen. (**F**) Increasing of vascular permeability of cutaneous blood vessels in mice triggered by LiRecDT1. (**G**) Dermonecrosis and histopathological changes following LiRecDT1 injection in rabbits’ tissue. (I) 5 µg of LiRecDT1 was injected subcutaneously in back skin. Arrow shows the site of injection and arrowheads show spreading of dermonecrotic lesion after 24 h. (II–V) Histopathological findings of rabbits’ skin 24 h following LiRecDT1 exposure. (II) Arrows show necrotic sites; (III) massive inflammatory response into the dermis and disorganization of collagen fibers pointed by arrows; (IV) massive inflammatory cell accumulation within dermal blood vessels (arrow); (V) hemorrhagic sites into the dermis are pointed by arrows. (**H**) Time-dependent direct hemolysis activity in rabbit erythrocytes treated with Phospholipase D of *Loxosceles gaucho*. (**I**) Cartoon representation of the structures of Brown spider venom PLDs: structural features highlighted in green, cyan and orange (catalytic, flexible, and variable loops, respectively) are from *Loxosceles intermedia* (PDB code: 3RLH) and in red are from *Loxosceles laeta* (PDB code: 1XX1). Amino acids participating in binding to Mg^2+^ (green sphere), catalysis and disulfide bridge formation are included in atom colors. (**J**) Mg^2+^ coordination (green sphere) by amino acid side chains and solvent molecules. Bound sulfate ion (yellow, red) is included. All procedures involving animals were carried out in accordance with “Brazilian Federal Laws”, following the Institutional Ethics Committee for Animal Studies Guidelines from Federal University of Paraná (Certificate n° 1112 of the Federal University of Paraná).

Despite the complexity of the venom, both native and recombinant PLDs alone can reproduce virtually all the clinical symptoms of Loxoscelism. Although the PLDs are not the most expressed toxins in *Loxosceles* venoms, they are the best studied and characterized toxins. Figure 1 summarizes the main aspects and activities of Brown spider PLDs by displaying updated assays and analyses performed for this work. The SDS-PAGE profiles of *L. intermedia* venom and a recombinant PLD are shown in Figure 1B and reveal an enrichment of proteins in the region of 30 to 35 kDa when venom is analyzed and a single band near to 35 kDa when the recombinant PLD was assayed. When amino acid sequences of representative PLDs from different *Loxosceles* species are aligned (Figure 1C), it is possible to observe the conservation of the residues involved in catalyses, as well as the preserved position of cysteine residues. The cross-reactivity of recombinant PLDs with native toxins of whole venom is demonstrated by a positive reaction in ELISA (Figure 1D), which suggests the conservation of epitopes between native and recombinant PLDs. Various biological activities of recombinant PLDs were brought together by updated assays whose results are depicted in Figure 1E–H. Figure 1E illustrates the insecticidal activity of a recombinant *L. intermedia* PLD, which was already described for a recombinant PLD of *L. arizonica* [12]. Unlike animals of control group, insects that received recombinant PLD were paralyzed 15 min after exposition (turned upside down in images), and no recovery was observed. These crickets were dead within 24 h. The increase in vascular permeability and development of dermonecrosis by *Loxosceles* PLDs demonstrated before [13,14,15,16] were reinforced here by the results of the biological assays showed in Figure 1F,G. The massive inflammatory response, necrosis and interstitial edema found in the histological analyses of rabbit skin exposed to a *L. intermedia* PLD (Figure 1G II–V) corroborate with those previous studies and agree with the histopathological changes triggered by the whole venom [17]. In addition, when the structures of the catalytic, flexible, and variable loops of *L. intermedia* and *L. laeta* PLDs are overlapped (Figure 1I), it is possible to observe that the main differences lies in the position of the flexible loop, since this *L. laeta* PLD naturally lacks the Cys53/Cys202 disulphide bridge, which is present in the *L. intermedia* PLD, as previously reported [18].

## 2. Nomenclatures and Biochemical Classification of Brown Spider PLDs

Since the main symptoms of accidents caused by spiders of the genus *Loxosceles* are skin lesions, with necrosis of the all envenomed tissue structures, Loxoscelism is also referred to as Cutaneous arachnidism or Gangrenous arachnidism [20]. The existence of a putative toxin involved in necrosis following accidents with spiders of the genus *Loxosceles* was initially postulated as Necrotic Toxin or Dermonecrotic Toxin [21,22]. Following the discovery in the venom of *Loxosceles reclusa* of a toxin capable of cleaving sphingomyelin, producing ceramide-phosphate and choline, involved with hemolytic activities and platelet aggregation, this molecule was then called Sphingomyelinase-D [23,24,25]. Over the next few years, the two terms *dermonecrotic toxin* and *sphingomyelinase-D* were used to designate the same molecules involved in some deleterious effects observed following envenomation by spiders of the genus *Loxosceles*.

In 2005, Machado et al. [26], using mass spectrometry and bi-dimensional electrophoresis, characterized 11 isoforms of toxins from venom of the South American species L. gaucho and since these toxins were involved in skin necrosis, the authors referred to it as Loxnecrogin. In the same year, Lee and Lynch [27], working with a recombinant toxin from the Loxosceles reclusa venom, showed that this toxin, besides cleaving sphingomyelin, was able to cleave other phospholipids such as lysophosphatidylcholine, lysophosphatidylinositol, lysophosphatidylserine, lyso-PAF and cyclic phosphatidic acid. The authors argued that because of this broad phospholipase activity exhibited, the term sphingomyelinase-D was very limited, and proposed the term Phospholipase-D [27]. Further studies confirmed these data by showing that recombinant PLD toxins from L. intermedia venom cleave sphingomyelin, lysophosphatidylcholine, and lyso-PAF [28,29], and that a recombinant PLD toxin from L. arizonica venom presented activity on sphingomyelin, lysophosphatidylcholine and lysophosphatidylethanolamine [30]. Since these enzymes from different species of Loxosceles have the ability to hydrolyze a wide range of different phospholipids, they are now classified as Phospholipases-D [5,30]. In an attempt to standardize the nomenclature of these toxins, a classification was proposed based on the amino acid sequence alignment of the various PLDs present in the Loxosceles intermedia venom deduced from cDNA sequences, phylogenetic studies and also on the biochemical and biological properties of these molecules. This study showed that, although all toxins possess the signature of the catalytic site of PLDs, some did not display all the biochemical activities such as enzymatic activity on sphingomyelin, or dermonecrotic activities in rabbits. The authors suggested using the definition of the Loxtox Family, pointing out the existence of six distinct groups [31]. Based on similar parameters and on the classification of spiders of the genus Loxosceles as members of the Sicariidae Family, the broader term of SicTox was proposed to represent toxin members of Brown spider venoms [32,33,34]. Currently, although all attempts to classify homologues of the Loxosceles genus spider venom toxins with phospholipase-D activities or amino acid similarities are valid, what is usually employed are the nomenclatures based on the biochemical properties of these enzymes, which act by cutting a wide variety of phospholipids (Phospholipases-D or PLDs) or by preferentially cutting sphingomyelin among different phospholipids (Sphingomyelinases-D).

## 3. The History of PLDs in Loxoscelism and the Production of These Toxins as Recombinant Proteins

The first descriptions of the clinical signals of the accidents did not detect the presence of a sphingomyelinase-D. However, in the middle of the 20th century, Atkins et al [22]. suggested that the venom of *Loxosceles reclusa* contained a toxin with necrotic activity on different tissues. Subsequently, some studies were published showing hemolytic and cytotoxic activities of venom [35,36]. Histopathological analyzes of envenomed tissues samples indicated a crucial role of polymorphonuclear (PMN) leukocytes (segmented neutrophils) in the dermonecrotic lesion caused by the venom of *Loxosceles reclusa* [37]. Nevertheless, Majeski et al. [38] affirmed that the venom directly causes inactivation of leukocytes. These authors suggested that neutrophils moved to the lesion area due to the activation of complement sequence by the necrotic tissue, based on a previous study that characterized the venom as an inhibitor of the complement sequence [39]. A few decades later, using cell culture techniques, Patel et al. [40] showed that the activation of the PMN leukocytes, which participate in necrotizing activity during the envenomation, is due to an indirect activation caused by toxins of *L. reclusa* venom on endothelial cells of the blood vessels, which in turn, activate the PMN leukocytes.

The first biochemical data in the literature pointing out the existence of a toxin characterized as sphingomyelinase-D in venom of a *Loxosceles* spider was published in 1978. Forrester et al. [23] showed that a purified fraction of the venom of *L. reclusa*, capable of causing lysis of sheep and human erythrocytes, cleaved sphingomyelin, producing ceramide-phosphate and choline, thus being characterized as a Sphingomyelinase-D. Later, Kurpiewski et al. [24] purified a *L. reclusa* sphingomyelinase D, and showed that the platelet aggregation effect caused by the venom of *L. reclusa* was also dependent on sphingomyelinase-D activity. This effect was later shown to be dependent upon the presence of serum amyloid protein [25]. Sphingomyelinases D from the venoms of *L. gaucho*, *L. laeta* and *L. intermedia* were also purified in the following years and featured as dermonecrotic, hemolytic and lethal toxins [41,42,43,44]. With the exception of these few studies, the majority of investigations concerning the activities of sphingomyelinases D were carried out using crude venom of *Loxosceles* species until the beginning of the 21st century, when the first descriptions of production of these toxins in the recombinant form were made. 

Many groups reported the production of the first recombinant toxins characterized as PLDs of *L. intermedia* [45] and *L. laeta* [46]. Lee and Lynch et al. [27] reported the recombinant expression of a PLD from *L. reclusa*. Other expressions of recombinant PLDs from the Loxosceles venoms characterized the existence of a family of isoforms/homologues of PLDs within the same venom (intra-species). For instance, to date, 8 PLD homologues from the *L. intermedia* venom have been expressed (Table 1), all containing the same amino acids involved in the catalysis, but varying in the primary structure of the proteins and in the biochemical and biological activities [13,14,16,45,47,48]. These data describing the existence of a family of PLD homologues within Loxosceles intra-species venom were confirmed by proteomic analysis and immunological assays using poly and monoclonal antibodies against a recombinant PLD, which recognized several proteins analyzed by bi-dimensional criteria (IEF and SDS-PAGE) [26,49,50]. Other recombinant PLDs from venoms of different *Loxosceles* species have been described in *L. laeta* [18,51,52], *L. reclusa* and *L. boneti* [51], *L. arizonica* [12,30] and *L. gaucho* [53]. They were all produced in prokaryotic expression systems with different tags. Table 1 describes these recombinant toxins and their main features.

## 4. Studies with *Loxosceles* Venom PLDs Proved Their Participation in the Physiopathology of Envenoming

**Figure 2:**
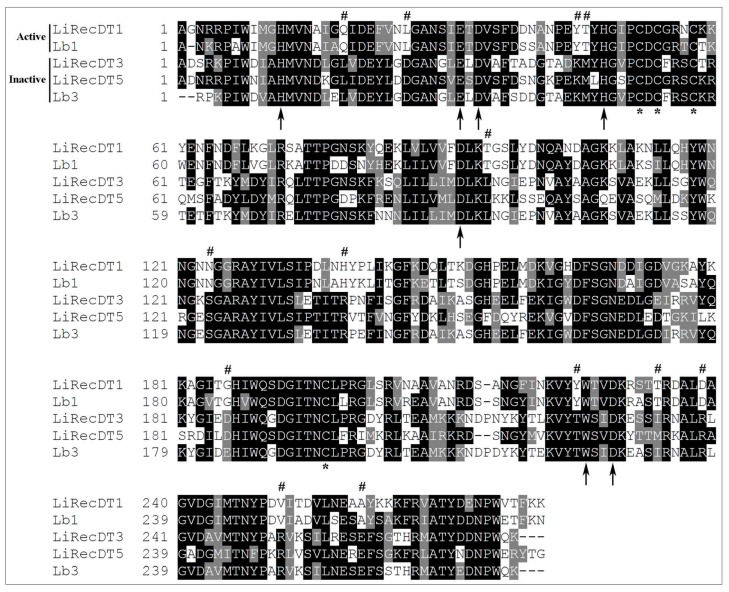
Enzymatically active and inactive Brown spider venom PLDs. Multiple sequence alignment of phospholipases-D of *Loxosceles intermedia* (Li) and *L. boneti* (Lb) venoms: LiRecDT1 (GenBank accession number ABA62021)*,* Lb1 (AY559844)—active PLDs; LiRecDT3 (ABB71184), LiRecDT5 (ABD91847) and Lb3 (AAT66074)—inactive PLDs. Sequences were aligned using the CLUSTAL X2 program [19]. Amino acid identities are shaded in black. Conservative substitutions are in gray, arrows point to amino acid residues involved in catalysis. The asterisks show cysteine residues. Hashtags indicate amino acids in active enzymes that are replaced by amino acids with different side chains in inactive isoforms.

The participation of PLDs of Brown spider venoms in the physiopathology of Loxoscelism is well-defined, and was strengthened with the advent of recombinant forms of PLDs obtained from different species of spiders. As outlined in Table 1, the majority of recombinant *Loxosceles* PLDs possess in vitro sphingomyelinase activity, except for LiRecDT3 (*L. intermedia*), LiRecDT5 (*L. intermedia*), LlPLD2 (*L. laeta*) and Lb3 (*L. boneti*). LiRecDT3 and LiRecDT5 isoforms do not induce dermonecrosis and platelet aggregation. In addition, LiRecDT3 do not cause mouse mortality, have small edematogenic activity and low antigenic potential. LlPLD2 and LiRecDT3 have low hemolytic activity [14,15,51,52], although LlPLD2 was expressed without the His 12 residue, which was shown essential for the catalytic activity of these enzymes. LiRecDT3, LiRecDT5 and Lb3, however, maintain the amino acids involved in catalysis, but have some important variations in residues neighboring the catalytic site, which could explain the lack of enzymatic activity, as hypothesized earlier [14,15,51,52]. Figure 2 depicts the alignment of these three enzymes that do not have sphingomyelinase activity and active PLDs of the same species (i.e., *L. intermedia* and *L. boneti*). It is possible to observe the conservation of amino acids involved in catalysis in all sequences, but also many non-conservative substitutions among active and inactive PLDs. The amino acids in active enzymes that are replaced by amino acids with different side chains in inactive isoforms are pointed out in the Figure.

All the recombinant PLDs that possess in vitro sphingomyelinase activity, from different *Loxosceles* species, are able to trigger the main symptoms observed following envenoming by *Loxosceles* venoms. For instance, we can list local symptoms such as the development of dermonecrosis, massive inflammatory infiltrate, increase in vascular permeability, and edema. In addition, systemic disturbances, such as hemolysis, platelet aggregation and nephrotoxicity are also reported after exposure to recombinant PLD. A variant isoform of LiRecDT1 containing a single H12A mutation at the enzyme catalytic site lacks enzymatic activity and is only able to trigger a residual inflammatory response. This outcome is most likely due to this substitution, which directly impairs the catalytic activity. Other interesting data showed that this mutated PLD with the H12A substitution did not cause dermonecrosis in the skin of rabbits nor direct hemolysis in human erythrocytes [28].

Different PLDs from *L. intermedia* and *L. gaucho* induce platelet aggregation in platelet rich plasma, and this activity also appears to depend on their enzymatic activity [13,14,48,53]. Direct and complement (C)-dependent hemolysis are also functional activities of *Loxosceles* PLDs. Isoforms 1 and 2 of *L. intermedia* PLDs (Loxtox groups 1 and 2) can induce direct hemolysis [13] and complement dependent hemolysis [54]. *L. gaucho* PLD similarly trigger direct hemolysis [53]. C-dependent hemolysis is also induced by SmaseD from *L. reclusa* [27] and by Smase I, LlPLD1 (similarity of 96% between Smase I and LlPLD1) and Smase II, all recombinant PLDs of *L. laeta* [18,46,52]. Some authors that report C-dependent hemolysis relate this activity to the removal of glycophorins by endogenous metalloproteinases activated by sphingomyelinase activity of PLDs and, thus, facilitating complement activation and hemolysis [18,54]. Direct hemolysis is also dependent on the bioactive products generated by the metabolism of membrane phospholipids after enzymatic activity [29,55]. It is important to mention that kidney injury caused by *Loxosceles* venom might be related to hemolysis. However, involvement of PLDs from Brown spider venoms in renal injury was brilliantly demonstrated by the use of wild-type PLD (LiRecDT1) and the mutated isoform with a substitution in the catalytic domain (LiRecDT1H12A). Treatments of mice with LiRecDT1, but not with the mutated toxin, evoked histological changes in the glomeruli and renal tubules of treated animals, increased blood urea and proteinuria, and caused animal lethality [59].

The role of Brown spider venom PLDs in the pathophysiology of envenomation was indirectly shown by the neutralization of the lethality of venom preparations in mice, using an antiserum produced with recombinant PLDs. The polyclonal sera were obtained by the inoculation of PLD recombinant isoforms from *L. laeta*, *L. reclusa* and *L. boneti* venoms in rabbits and horses [51]. A rabbit antiserum, produced with a recombinant PLD of *L. laeta*, significantly reduced the dermonecrotic lesion in rabbits challenged with the venom preincubated with serum [46]. More recently, Oliveira et al. [60] showed that mice serum produced against a recombinant PLD of *L. intermedia* inhibited dermonecrosis, hemorrhage and edema in rabbits challenged with the same toxin preincubated with serum.

Finally, using constructs with site-directed mutations, several homologous of recombinant forms of PLD toxins from *L. intermedia* venom were studied. The data not only confirmed the participation of PLDs in the pathological picture of the envenomation, reproducing dermonecrosis, hemolysis and increased capillary permeability, but also indicated the role of several specific amino acid residues in the catalytic mechanism responsible for the development of these effects [61]. In summary, it is safe to say that the data described in the literature are quite strong and prove the participation of *Loxosceles* venom PLDs as molecules involved in the dermonecrotic, inflammatory, hemolytic, nephrotoxic and thrombocytopenic effects described in the injured patients.

## 5. Structural Organization and the Catalytic Mechanisms of Brown Spider PLDs

The *Loxosceles* venom PLDs are impressive molecules, considering their biochemical and biological activities [2,5]. These enzymes cleave phospholipids in the bond between the phosphate and the hydrophilic moieties. Activities of PLDs have been described on various phospholipids in biological membranes, such as sphingomyelin, lysophosphatidylcholine, lysophosphatidylserine, lysophosphatidylinositol, cyclic phosphatidic acid, lyso-PAF, among others examples [27,29,30]. Undoubtedly, the great development regarding structure and mechanism of PLDs from *Loxosceles* genus spiders has been obtained with the production of these enzymes in the recombinant form. Different PLDs from *Loxosceles laeta*, *L. intermedia* and *L. gaucho* have been crystallized and their three-dimensional structures have been determined at high-resolution and combined with in silico analysis, docking and molecular dynamics we now have a better understanding of the precise steps involved in the catalytic mechanism of these enzymes. These PLDs contain between 284 and 285 amino acid residues, and are organized as a barrel formed by 8 parallel beta strands linked to alpha helices, resulting in a general fold referred to as (alpha/beta)_8_ or a Triose Phosphate Isomerase (TIM) barrel. PLDs share structural homology with TIM, an enzyme with a similar three-dimensional fold [5,62,63,64,65]. The amino acid residues involved in the catalytic activity of these PLDs (His12, Glu32, Asp34, Asp91, His47, Asp52, Trp230, Asp233, and Asn252) [62] are well conserved in all isoforms of *Loxosceles* PLDs identified to date [5,61]. Structural analysis based on the crystal structures and molecular dynamics, showed that His12 and His47 residues are directly involved in the catalytic activity of PLDs [18,62,66]. His47 functions as the nucleophile initially acting on the phosphodiester linkage to release choline and His12 participates in the formation of ceramide-1-phosphate, which is the final product generated by the action of the PLDs on sphingomyelin [62]. The residues of the amino acids glutamic acid (Glu) 32, aspartic acid (Asp) 34 and 91 are involved in the coordination of a Magnesium ion (Mg^2+^). In addition, Mg^2+^ plays an important role in the recognition and binding of lipid substrates cleaved by PLDs, and also plays a role in the stabilization of the enzyme-substrate complex [5,62,65]. Although sphingomyelins and lysophosphatidylcholines have been shown to be the preferred substrates for PLDs, the precise steps in the interaction with lipids is still not completely understood. Several reports argue that the *Loxosceles* venom PLDs display hydrolytic activity on their lipid substrates and cleave the phosphodiester bond in phospholipids, generating ceramide-1-phosphate when sphingomyelins are hydrolysed and lysophosphatidic acid when lysophosphatidylcholine is hydrolysed [2,5,27,65]. However, as previously shown for several other non-loxoscelic PLDs, these enzymes can mediate transphosphatidylation reactions. Nuclear magnetic resonance (NMR) and mass spectrometry (MS) data demonstrate that these enzymes generate cyclic lipids by transphosphatidylation of its phospholipid substrates, producing cyclic ceramide-1-phosphate when acting on sphingomyelins, and cyclic phosphatidic acid when acting on lysophosphatidylcholines [67]. This new model requires a new interpretation in the molecular mechanisms of these enzymes [30]. According to Lajoie et al. [30], this mechanism could be similar to the bacterial enzyme GDPD (glycerophosphoryl phosphodiesterase), which also generates cyclic lipids following its catalytic action on phospholipids. In this model, the residues of amino acids H12 and H47, which are conserved in all loxoscelic PLDs described so far, would play an important role in catalysis. These authors also propose another molecular model for the mechanism of *Loxosceles* PLDs (based on molecular dynamics and docking models), in which there are some differences in the orientation of phosphoglycerol groups from the GDPD model. In this model, called “Reverse Orientation Model”, the hydrophilic part of the phospholipids (the polar head) would be oriented into the binding pocket of the PLD, where H47 would act as a nucleophilic group, whereas H12 could protonate the cleaved polar group. The Mg^2+^ ion would act by accelerating the catalytic reaction resulting in the stabilization of negative charges generated in the system. The structures of recombinant PLD isoforms containing mutations H12A [28,49,59] and H12A-H47A, D34A-E52A [61] confirmed the crystallographic data and indicated the role of these amino acids in the catalytic activity of these enzymes. In addition, the Y228A isoform of PLD of *L. Intermedia,* resulted in the wild-type active form turning into an isoform with only residual activity for both the catalysis of sphingomyelin and for biological effects such as hemolysis and dermonecrosis [61]. Interestingly and corroborating this hypothesis, the inactive isoforms of PLDs LiRecDT3 and LiRecDT5, from *L. intermedia*, and Lb3, from *L. boneti*, present a modification in this region Y229T (Figure 2) [13,14,15,51]. These PLDs display only residual activities for dermonecrosis (*L. intermedia* isoforms) and catalysis (all isoforms). Another experimental data that strengthens the importance of the aromatic triple region (Y228-Y229-W230) in the *Loxosceles* venom PLDs was demonstrated by substitution of tryptophan (W230A), which resulted in an enzyme that neither degraded sphingomyelin nor possessed hemolytic activity [68]. This same study showed that the mutation D259G (corresponding to D233 when numbering excludes amino acids from the signal peptide) produced loss of activity upon sphingomyelin, and mutation S262A (corresponding to S236) caused a decrease in the enzymatic activity upon the same substrate.

In addition, mutant versions of the enzyme supported the understanding of the binding to their lipid substrates, especially with choline-containing phospholipids, such as sphingomyelin and lysophosphatidylcholine. In this sense, the region containing three aromatic amino acid residues Y228, Y229, and W230, which are conserved in all the isoforms of *Loxosceles* venom PLDs with high catalytic and biological activities, has been postulated as playing an important role in the binding and orientation of the substrate relative to the catalytic site [6]. The explanation for such a change in these activities would be that, in the wild-type form, the region rich in aromatic residues (Y228, Y229, and W230) would interact with the hydrophilic head of the substrates by cation-π bonds, with emphasis on the choline, as previously shown for other PLDs [69,70]. 

**Figure 3:**
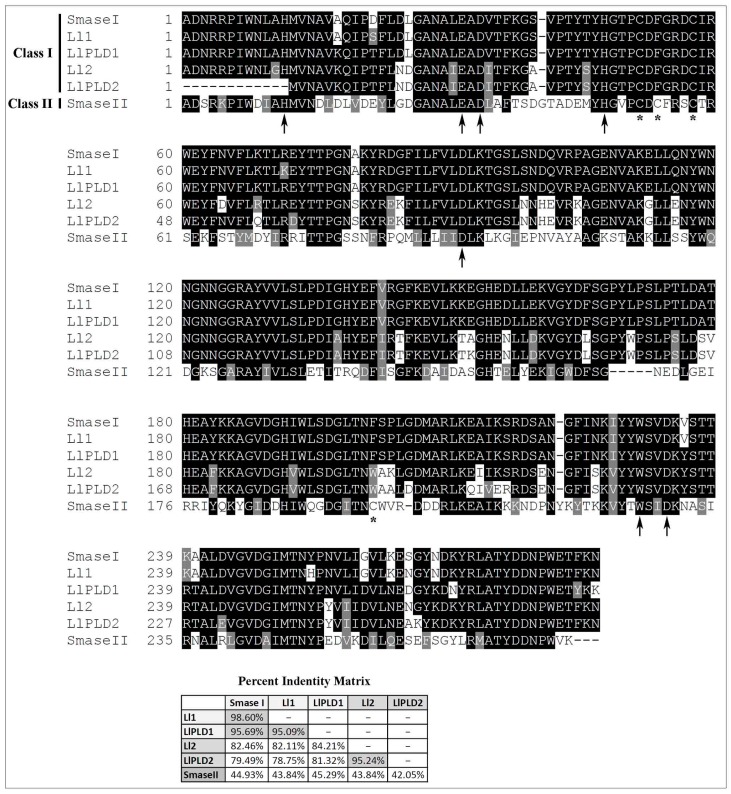
*Loxosceles laeta* Class I and II recombinant PLDs (A) Multiple sequence alignment of *L. laeta* class I phospholipases-D Smase I /LlSicTox-alphaIII1i (GenBank accession number AY093599), Ll1/LlSicTox-alphaIII1ii (DQ369999), LlPLD1 (GU121905), LlPLD2 (GU121906), Ll2/LlSicTox-alphaIII2 (DQ370000) and class II Smase II/LlSicTox-betaIA1 (AY093601). Sequences were aligned using the CLUSTAL X2 program [19]. Amino acid identities are shaded in black. Conservative substitutions are in gray, arrows point to amino acid residues involved in catalysis. The asterisks show cysteine residues. (B) Percent identity matrix showing the similarities among aligned PLDs. Values given by CLUSTAL X2 program [19].

Analyzes of the three-dimensional structures of these PLDs show a similar spatial organization containing three conserved regions, which were denominated catalytic, flexible and variable regions. The presence of one or two disulfide bonds formed by the cysteine residues (Cys51-Cys57 and Cys53-Cys201) stabilizes the catalytic site and controls the size of the catalytic cleft of *Loxosceles* PLDs [62]. According to this organization, it was suggested a classification of these enzymes in Class I, for those PLDs containing only one disulfide bridge maintained by the residues (Cys51 and Cys57), and Class II, for those PLDs containing the two disulfide bridges [63]. Experiments using a recombinant isoform with mutations in the second disulfide bridge (Cys53A-Cys201A) of a Class II PLD show that the variant obtained has its catalytic and biological activities reduced, but not blocked [61]. However, the presence or absence of the disulfide bridge between Cys53 and Cys201 is not by itself a feature that defines the enzymatic performance of a *Loxosceles* PLD. As shown in Figure 3, most *L. laeta* isoforms belongs to Class I, but Smase II possess the four cysteine residues that form the two disulfide bonds, being then classified as a Class II PLD. As described in Table 1, all the recombinant *L. laeta* PLDs are enzymatically active, except for LlPLD2 that lacks one histidine of the catalytic site.

## 6. Proteomic Analysis in the Learning about Brown Spider Venom PLDs

Proteomic studies participated in the description of advances in learning about *Loxosceles* PLDs. The first proteomic analysis of a Brown spider venom was performed by Cunha et al. [71], who studied the venom of *Loxosceles gaucho* by means of liquid capillary chromatography and mass spectrometry. This work identified two toxins with approximately 31.4 and 31.6 kDa belonging to the sphingomyelinase-D family. In this same year, Binford and Wells [72] published a proteomic analysis by mass spectrometry of the venoms of 10 different species of spiders from *Loxosceles* genus (*L. arizonica*, *L. deserta*, *L. apachea*, *L. alamosa*, *L. reclusa*, *L. rufescens*, *L. laeta*, *L. speluncarum*, *L.* sp. Hoogenoeg, and *L. spinulosa*), describing the presence of toxins with molecular masses between 31 and 35 kDa, which were characterized as sphingomyelinases-D in all venoms. This work confirmed the idea of a family of these toxins in different Brown spider venoms (inter-species) and within the same venom (intra-species), since the study also showed the presence of more than one molecule in the mass range of sphingomyelinases-D within the same species. Additionally, Machado et al. [26] analyzed the venoms of *Loxosceles intermedia*, *L. laeta* and *L. gaucho* (three South American Brown spider species), using several techniques, such as two-dimensional electrophoresis, amino acid sequencing, Edman degradation, immunostaining and mass spectrometry. Data obtained classified at least eight toxins as sphingomyelinases-D isoforms in the three different venoms, strengthening the results regarding the existence of an intra-species and inter-species families of these enzymes. In addition, *L. gaucho* venom was fractioned and studied by mass spectrometry, which enabled the detection of 11 isoforms of toxins similar to sphingomyelinase-D. At this time, different venoms of *L. intermedia*, *L. gaucho*, *L. similis* and *L. adelaida* were analyzed by means of two-dimensional electrophoresis, showing a profile of proteins with enrichment in the region of 30-35 kDa and isoelectric points among 4 to 10, which are compatible of sphingomyelinases-D [73,74,75].

In 2009, toxins from *Loxosceles intermedia* crude venom were identified using a MudPIT shot-gun approach, and the data confirmed the presence of peptides with homologies to sphingomyelinases-D [76]. In addition, a comprehensive profile of *Loxosceles intermedia* crude venom toxins was obtained by the digestion of the venom followed by the separation of fragments using UHPLC liquid chromatography and analysis by mass spectrometry (ORBITRAP). The final data showed the presence of peptides compatible with different members of the PLD family in the venom, confirming the existence of a large intra-species family of these molecules [77].

## 7. Cellular Biology of the Brown Spider Venoms PLDs

If on the one hand, the molecular structure and mechanism of Brown spider venom PLDs are reasonably well understood, the cellular biology of envenoming still requires much more learning and molecular detailing. Concerning the pathophysiology of envenoming, literature accepts that Brown spider PLDs trigger their toxic effects evoking an unregulated inflammatory response caused by an uncontrolled activation of neutrophils by endothelial cells, leading to necrosis of tissues [2,5]. The PLDs act on membrane phospholipids or even soluble substrates in tissues of the exposed animals (sphingomyelins and/or lysophosphatidylcholines), generating biologically active lipids, such as ceramide-1-phosphate (C1P) and/or lysophosphatidic acid (LPA), which trigger an endothelial cell-dependent activation of leukocytes, causing an exacerbated inflammatory response and tissue necrosis [78,79]. However, there is no data to sustain if these lipids could be further transformed into the intra- or pericellular environment by the action of kinases, phosphatases or other enzymes, in order to produce derivatives such as ceramide, sphingosine and sphingosine-1-phosphate from the C1P, as well as phosphatidic acid from LPA. These molecules are also biologically active lipids and important actors involved in leukocyte activation and uncontrolled inflammatory response [79]. It is also not known whether there is an internalization of PLDs by the cells at the bite site region, and subsequent generation of biologically active lipids within the cells, or if lipids generated at the extracellular side of cell membranes by PLDs activity could be translocated into the cells by the change in their hydrophobic coefficient, loss of polar heads and/or phosphate, and then activate cascades of intracellular signaling. Alternatively, lipids generated outside the cells could activate G protein-coupled receptors on plasma membranes. In addition, it has been recently described that these PLDs can generate cyclic lipids such as cyclic ceramide-1-phosphate and cyclic phosphatidic acid [67]. Would these cyclic molecules act directly, or would they be complexed to cellular receptors? Would they be internalized in cells, or would they be transformed into non-cyclic derivatives? These are unanswered questions in the field at this time. 

Moreover, concerning the cellular environment of the tissues affected by the PLDs, it is not known which cells are the direct targets of these enzymes. The cellular biology involved in the action of Brown spider PLDs is believed to be related to their actions on endothelial cells [5,28,40], but literature data also point to the role of fibroblasts [80,81], platelets [13,14,48] and erythrocytes [29,82], which could be related to the pathophysiology of envenoming. Currently, there is very little data available that describes how Brown spider venom PLDs would interact with cell membranes. There is no information about if or how these toxins alter the biophysical properties of cell membranes. 

The ability of PLDs to interact with the plasma membrane was shown in human red blood cells treated with a PLD of *Loxosceles intermedia* venom. Results showed these enzymes could bind to the surface of erythrocytes stimulating the formation of lipid aggregates, and in some cases disturbing the integrity of these structures. The interaction between PLDs and the surface of cells was also demonstrated by human red blood cells treated with the chimera GFP-PLD, a recombinant PLD fused to a green fluorescent protein (GFP). The linkage could be visualized by the intense cell labeling [82]. These data were reproduced by incubating B16-F10 cells (murine melanoma) with the same chimera (GFP-PLD) and noticing the intense labeling of cells [49]. Alternatively, cells were exposed to PLD, which was further immunodetected by using the respective antibody. However, unlike the erythrocytes that were lysed after treatment with the PLDs, B16-F10 cells did not show any sign of cytotoxicity, suggesting that, in some way, the differences in the composition or organization of membrane lipids on the cells regulates the cytotoxicity of these toxins [49]. 

The effects of PLDs on artificial membranes were also evaluated using a *Loxosceles laeta* recombinant PLD. The main conclusions were that: the activity of this PLD is clearly influenced by the supramolecular organization of its substrate in the target membrane; the transformation of the target phospholipids (sphingomyelin) into lipid derivatives in the membranes alters the structure and morphology of the membrane, promoting the membrane fusion and generating non-lamellar structures. When recombinant PLD from *Loxosceles laeta* was incubated with giant fluid unilamellar vesicles composed of sphingomyelin containing acyl groups with 12 Carbon (C12SM), it could be observed membrane lipid disorganization, lipid aggregation, tubular projections and destruction of vesicles. The presence of cholesterol in membranes alters and decreases the activity of PLD [83]. The explanation could be linked to the role of cholesterol impairing the interaction between the PLD and SM by changing the transition phase of the membrane. In the case of the vesicles containing more than one substrate, for instance phosphatidylcholine:sphingomyelin:colesterol, the action of the PLD is different, causing neither the destruction of vesicle, nor the tubular projections, but altering the initial distribution of the substrates in the vesicle. Thus, the effects of PLD enzymes were dependent on lipid composition in the different vesicles tested, explaining why different cells would undergo different actions by PLDs [83].

## 8. A New Generation of Anti-loxoscelic Sera and Vaccines using *Loxosceles* Recombinant PLDs and Synthetic Peptides

Treatment for victims of Brown spider bites may be symptomatic, based on drugs that seek to block the exacerbated inflammatory response in injured areas. In this case, anti-inflammatory drugs such as dapsone, analgesics and corticosteroids are used. Antihistamines and antibiotics may also be prescribed depending on the presence of allergic reactions and secondary infections in the necrotic lesions of the skin. Procedures that seek to reduce tissue necrosis damage, such as oxygen therapy in hyperbaric chamber and surgical procedures, are also indicated [4,5,84,85]. The only specific treatment, which aims to neutralize venom toxins, is the serum produced in horses using crude venom of Brown spiders. The antigenic conservation among venom toxins from different spider species of *Loxosceles* genus was shown by immunological-cross reaction between sera developed against a particular venom and toxins of other venoms. For example, a serum developed against the crude venom of *Loxosceles gaucho*, which recognizes toxins from *L. laeta* and *L. intermedia* venoms [43]; or a serum produced with the *L. reclusa* venom that also recognizes toxins from the venom of *L. deserta* [86]. Antisera used for Brown spider envenoming treatments are available in countries such as Brazil, Argentina, Mexico and Peru. In Brazil, venoms of the three species of spiders with greater clinical significance are used (*Loxosceles intermedia*, *L. gaucho,* and *L. laeta*) [87]. However, the treatment of loxoscelism based on serum therapy is still controversial, since its efficacy varies according to the different types of accidents, the species involved and mainly the delay in the beginning of the treatment after the bite [2,3,5,85,87]. This variation in the efficacy of antivenoms also happens with some snake antivenoms, mainly regarding neutralization of venom activities from different species of the same genus, and authors suggest the use of a greater antivenom:venom ratio to circumvent this problem [88]. However, the lack of effectiveness of these anti-loxoscelic sera could be related to their low titers of anti-PLD antibodies, and thus fail to neutralize these toxins after envenoming. The discoveries of venom PLDs pointed these toxins as potential targets to be used as antigens for a new anti-loxoscelic serum. They could also be used as antigens to enrich the crude venoms and then generate anti-venom sera with possible better therapeutic response. Three facts strengthen these hypotheses: (i) sera against crude venoms or venom gland homogenates of different species of Brown spiders recognize different members of the PLD family, and neutralize the main effects of these venoms [43,89,90]; (ii) monoclonal antibodies produced against native or recombinant PLD toxins were able to recognize several isoforms of PLDs from different species and neutralize the effects of crude venom or an homologous PLD [50,91]. (iii) sera produced against recombinant PLDs from *L. laeta* neutralize the necrotic and lethal effects of different *Loxosceles* venoms [46,51,52], and sera from mice immunized with a PLD from *L. intermedia* venom protected the animal against dermonecrosis, edema and hemorrhage [60]. 

However, three facts are relevant in the use of recombinant PLDs as antigens in a novel anti-loxoscelic serum: (i) native recombinant forms are aggressive and cannot be used in high concentrations, since even horses could have important side effects or die, thus restricting the production of anti-PLD antibodies. (ii) Although there is an immunological cross reaction and conservation of epitopes among PLDs of different species of spiders from *Loxosceles* genus, the ideal strategy for neutralization of the pathological effects observed in accidents would be to use a mixture of recombinant PLDs of the main species geographically involved in the accidents in each country or region (for instance in the case of Brazil and South America, *Loxosceles intermedia*, *L. gaucho,* and *L. laeta*; in the case of North America, *L. reclusa*, *L. deserta,* and *L. arizonica*). (iii) Other toxins in *Loxosceles* venoms may also act to increase the deleterious effects of PLDs, such as the so-called spreading factors. These toxins modify the organization of the extracellular matrix at the site of venom inoculation, and causes the disruption of adhesive cellular junctions, rendering the tissues looser by increasing capillary permeability and favoring the local and systemic spread of other toxins and host cells. Also, transcriptome analyzes and recombinant expression of toxins have pointed the presence of allergenic toxins in the venoms, a fact that could increase inflammatory response and facilitate the spreading of other toxins. In the case of *Loxosceles* venoms, at least three families of toxins with these characteristics were already described: LALPs (astacin-like metalloproteases) [92,93,94,95], LiTCTP (Translationally-Controlled Tumor Protein) family member with allergenic properties [96,97], and hyaluronidases [98,99,100].

In this sense, the development of a more efficient serum relies in studies that search for the relationship between structure and function, associated with site-directed mutation techniques [61,63,101,102] which described the amino acids involved in the catalysis and the binding of the *Loxosceles* PLDs with lipid substrates. Mutated variants of recombinant PLDs, previously described here, could be used as antigens in high concentrations in animals and give rise to a second generation anti-loxoscelic serum with higher antibody titers. Theoretically, this serum could neutralize in a more efficient way the deleterious effects of loxoscelic envenoming PLDs. On the other hand, these same mutated toxins could still be used as protective antigens in the construction of anti-loxoscelic vaccines, which could be indicated in endemic regions such as South America [2,5], or as veterinary use in domesticated animals, as well as in livestock, a quite common possibility [103]. 

It is also possible to use synthetic antigens containing antigenic determinants of toxins, which could generate antibodies that reduce the symptoms of envenoming. Linear synthetic peptides or mimotopes, based on a PLD sequences from *L. intermedia* venom, generated polyclonal antibodies that were able to block toxic effects evoked by the crude venom and/or recombinant PLDs [56,104,105]. In the same rationale, linear synthetic peptides, constructed based on PLDs sequences from *L. laeta*, *L. intermedia,* and *L. gaucho* venoms, are recognized by anti-venom sera from these spiders, indicating their potential as useful reagents to produce antibodies against *Loxosceles* venoms [106]. In addition, chimeras containing antigenic determinants of PLDs, LALPs and hyaluronidases could be synthetized to generate antibodies that act synergistically in the inhibition of envenoming. In fact, a synthetic chimera containing sequences of PLDs from *L. intermedia* and *L. laeta* venoms, and sequences of a LALP and hyaluronidase isoforms from *L. intermedia* venom was recently constructed. This fusion molecule stimulated the production of polyclonal antibodies which recognized venoms of the different species of *Loxosceles*, and blocked the activities of PLDs, metalloproteases and hyaluronidases present in the venoms [107]. 

In the near future, it is expected a new second-generation anti-*Loxosceles* serum and a protocol for vaccines using mutated recombinant PLDs from different species of *Loxosceles* alone or as antigen for crude venom enrichment, and also synthetic antigens containing antigenic determinants from different *Loxosceles* toxins. These molecules and strategies could be used to improve the specific treatment of loxoscelism, mitigating the suffering caused in several regions of the world where these accidents are endemic and recurrent. The use of recombinant forms of loxoscelic PLDs or synthetic peptides mimicking sequences of these enzymes was already considered an effective strategy in the generation of sera with high antibody titers, high neutralization capacity and therapeutic potential [108]. It is important to mention that these initiatives would not alter the production lines of conventional sera.

## 9. Brown Spider PLDs as Models for the Discovery of Specific Treatments for Loxoscelism and as Tools in New Anti-inflammatory Drugs Description

For all of its biological properties, pathological involvement and biochemical characteristics [2,5], Brown spider venom PLDs are important targets for inhibitory drug discoveries focusing on the clinical treatment of loxoscelism. These enzymes have also a great potential as modulators of various inflammatory processes, since they induce the formation of lipids which are involved in the cellular inflammatory response [5,30,109]. There is no data in literature regarding the molecular mechanisms of the intracellular signaling cascade activation in cells exposed to Brown spider venoms or their PLDs. Likewise, there are no data in the literature describing which molecules or intracellular signaling pathways are activated or blocked in cells exposed to PLDs of *Loxosceles* spider venoms to induce the exacerbated inflammatory response. If the action of *Loxosceles* PLDs on the membranes of injured host cells or surrounding fluids generates biologically active lipids, it is expected the participation of receptors of these phospholipid derivatives, such as those coupled to protein G, and the generation of intracellular secondary messengers, in addition to the activation of different genes [79,110,111,112]. 

In fact, it has been demonstrated that the treatment of cultured fibroblasts with a recombinant PLD from the venom of *L. reclusa* led to the activation of IL-6, IL-8, CXCL1, and CXCL2 pro-inflammatory genes [80]. In the same rationale, the secretion of these cytokines increased following treatment of human fibroblasts in culture with *L. similis* crude venom, or with a recombinant PLD of *L. intermedia* via the activation of lysophosphatidic acid receptors. On the other hand, when fibroblasts were treated with Ki16425, an antagonist of lysophosphatidic acid receptor, the release of these cytokines decreased [81]. In addition, the treatment these cells with recombinant PLDs from *L. laeta* venom increased the expression and secretion of IL-6 and IL-8 cytokines, and CXCL1/GRO-Alpha and CCL2/MCP-1 chemokines, which increased monocyte migration, and whose secretions were blocked by the use of anti-PLD antibodies in culture conditions [58]. Thus, these pathways are potential targets for drugs aiming loxoscelism.

Based on in silico analyzes, biochemical experiments, thermodynamic studies, structural analyzes and biological evidences on cells and animals exposed to a recombinant PLD from *Loxosceles intermedia* venom, it was shown that the drug suramin was able to inhibit various biological activities. Suramin, a former drug used in the treatment of protozoa and especially African Trypanosomiasis, blocked PLDs effects, such as hemolytic, necrotic and pro-inflammatory effects, as well as biochemical properties such as sphingomyelin degradation. Molecular dynamics and docking studies showed suramin interacts with the PLD catalytic site, especially with Histidine-12 and Histidine-47 amino acids residues, in addition to Mg^+2^ ion, involved in the enzyme catalysis [113]. Such pioneering studies opened the possibility of using suramin or pharmaceutical analogues (belonging to the polysulfonated naphthylurea group) as potential inhibitors of *Loxosceles* PLDs. 

It was also proposed the use of tetracycline in the treatment of loxoscelism, since cells exposed to *Loxosceles* venom PLDs increased the expression of extracellular matrix metalloproteases (MMPs). Tetracycline indirectly inhibits these proteases, thus blocking PLDs effects. Experiments with cells and animals exposed to a recombinant PLD from *Loxosceles intermedia* venom showed that tetracycline treatments were able to protect them from the dermonecrotic activity [114] and nephrotoxic effects [115] caused by envenoming. This data opened up the possibility to use tetracycline in the local and systemic treatment of loxoscelism. The knowledge in this field will not only contribute to advances in cellular mechanism of loxoscelism, but may also generate target molecules for the discovery of inhibitory drugs of receptor/signaling cascade involved in the lipid-dependent inflammatory response generated by *Loxosceles* venom PLDs.

## 10. Diagnosis of Loxoscelism Based on the Identification of PLDs or Related Products 

Although the involvement of Brown spiders in the necrotic picture presented by injured patients has been described for a long time [22], to date, there is not a single direct laboratory method for loxoscelism diagnosis. Nowadays, the diagnosis is mainly based on the capture of the spider shortly after the accident (which is not always possible) and its presentation in the Medical Centers for the correct identification, or on the epidemiology and natural geographic distribution of *Loxosceles* genus species (as is often the case in Southern Brazil), or even on the clinical signs and symptoms presented by the patients. The exclusion of other etiologies of dermonecrosis, although essential, are commonly a tough task for physicians [2,4,5]. Undoubtedly, the existence of a family of isoforms or homologs of PLDs in different *Loxosceles* venoms [5,6] confers to these molecules an enormous potential in the diagnosis of loxoscelism. At present, clinicians involved in the diagnosis and treatment of the injured patient can request skin biopsies from the regions of spider bite in inconclusive cases. The histopathological diagnosis is based on the characteristics described in experimental models (rabbits) exposed to the crude venoms of Brown spiders and/or native or recombinant *Loxosceles* PLDs [2,20]. 

The infrared thermography was proposed to aid in the diagnosis of loxoscelism, since it detects and quantifies the body surface temperature, which may reflect the presence of a huge inflammatory response at the site of the accident [116]. Although it is a non-invasive technique, the simple detection of an inflammatory response at the injured site does not necessarily reflect specifically accidents with Brown spiders and exposure to PLDs. There are reports of an increase in body surface temperature in several other medical conditions, including accidents with other venomous animals [116]. However, together with other signs and epidemiology of loxoscelism, this can be an interesting strategy, since it is a non-invasive method and there is a clinical correlation with injured victims. Several attempts have been done to identify toxins at samples from injured patients or experimental animal models inoculated with crude venom. Barrett et al. [117] proposed a haemagglutination inhibition test for the diagnosis of Brown spider accidents based on analyzes of skin samples of guinea pigs exposed to the venom of *Loxosceles reclusa*. Later, literature described an immunodiagnostic test (ELISA sandwich) capable of detecting toxins of the crude venoms of *L. intermedia*, *L. gaucho* and *L. laeta*, in mice inoculated with the respective venoms of these spiders [118]. In this same rationale of immunodiagnosis, Miller et al. [119] proposed a diagnostic method based on the competition ELISA technique, using as reagent a polyclonal serum that recognized crude venom. This study analyzed biopsies of skin lesions from patients envenomed by *L. deserta*. Additionally, a new method for the diagnosis of accidents, based on ELISA, was proposed using biopsies and fluids of skin lesions from injured patients [120,121]. Although all of these methods describe the potential to find toxins of *Loxosceles* venoms in the samples tested—even collected a few days after the accidents—none of them were unfortunately incorporated into the clinical routine of detection and treatment for the loxoscelism. This is most likely related to the low sensitivity of the method, the invasive aspect of sample collection, and the lack of clinical correlation. Alternatively, a noninvasive method was proposed in which samples of skin lesions from victims of *L. reclusa* were collected with swab moistened with water saline solution and analyzed by ELISA. The protocol used polyclonal antibodies that recognize toxins from the crude venom of *Loxosceles reclusa*. By this proposed method, the authors were able to recover and detect *Loxosceles* toxins in samples collected from the skin of different patients: approximately 34.0 picograms [122], between 4.5 and 8.2 picograms [123] or 190.0 femtograms of total toxins per ELISA well [124]. Still using this same method of immunodetection, tests were made to study the sensitivity of the method to recover and detect these toxins accordingly to the time after exposition to the venom. The authors used rabbits exposed to the crude venom as experimental model and swabs to collect skin samples. Toxins, including PLDs, could be recovered and detected up to 21 days after the exposure to the venom [125]. This method is not invasive and have clinical correlations of the patients with those described with loxoscelism; however, there is no data confirming that the reagents used would also be able to detect toxins of other species of *Loxosceles* spiders. In addition to that, the methodology is not available on the market for large-scale analyzes. 

Nowadays, with the advent of recombinant *Loxosceles* PLDs, ELISA methods availability and previous description, this immunodetection tests could be improved by the generation of monospecific polyclonal antibodies. This would allow PLDs recognition of different species of *Loxosceles* spiders, and also the possibility of using monoclonal antibodies for sandwich ELISA tests. Such methods, based on the search for PLD antigens in samples collected from skin lesions of victims, could be directed to these toxins by the high biological conservation of these molecules in the different venoms [5,6] and for their high determinant antigenic properties [107]. 

Although there are no published data in scientific literature, the diagnoses for accidents could also be based on other biological samples different from the lesions in bite sites. Urine tests could be performed for searching PLDs or their metabolized fragments, since envenoming causes renal damage and PLDs are directly involved with these lesions. It was already shown that PLDs bind to renal structures such as glomeruli and renal tubules, and thus could be excreted in the urine [47,59,73].

Methods for diagnosis of accidents with arachnids, among them Brown spiders, was a theme discussed by an article recently published. The authors discuss the limitations, propose techniques and recommendations that in the near future may originate laboratory methods for loxoscelism diagnosis involving detection of PLDs [126]. However, it has been recently described that patients who never had contact with spider venoms of the genus *Loxosceles* have antibodies that recognize PLDs from the venom of *L. laeta* and even other *Sicarius* spiders [127]. This could lead to false positive results in immunoassays such as ELISA and western blotting. These data indicate that other studies looking for further information on the antigenicity of these toxins need to be made, and the possible non-specific interference of these antibodies in immunoreactivity in laboratory diagnostics must be considered. 

Alternatively, the immunodiagnosis of loxoscelism could be done by using monoclonal antibodies that recognize PLDs from the *L. intermedia* venom, but manipulated by protein engineering, yielding a chimera containing a fragment of the IgG molecule bound to the alkaline phosphatase enzyme. Antigens from *Loxosceles* could be detected in the fluids of injured patients or samples collected at the spider bite site. A recombinant chimera was able to recognize PLDs from *L. intermedia* venom in immunoassays, with detection limits of up to 39 ng/mL [128]. 

In addition, highly sensitive molecular biology methods such as PCR could be used to look for DNA signals from PLD genes at the injured site. Venoms are holocrine secretions [129] and can contain cells from the venom-producing glands and their DNA. In this rationale, using two recombinant isoforms of the *L. laeta* venom, six RNA aptamers capable of inhibiting the phospholipase activity of these enzymes were described and, therefore, theoretically could be efficient in their identifications as a screening for possible diagnosis [130]. 

In summary, we already have, nowadays, a good knowledge regarding the components of the venoms, especially the PLDs. This should in a near future support a direct laboratory method capable of diagnosing accidents by Brown spider bites available for the medical community.

## 11. Biotechnological Applications of *Loxosceles* PLDs

The biotechnological use of venom toxins, and products of secretions of venomous animals, is a consolidated reality with several examples in the literature. There are several described products synthesized, based on molecular and biological properties of venom toxins from snakes, mollusks, bees, spiders, and scorpions—among other animals. The direct application of these toxins as biological reagents or drugs by pharmaceutical companies is also reported. In addition, there are several examples of the use of medicines based on venom toxins: in the treatment of hypertension, myocardial infarction, stroke, ischemia, diabetes, hemostatic disturbances, pain, neurological alterations, and autoimmune diseases, among others [131,132,133,134,135,136,137]. 

Undoubtedly, the PLDs of the *Loxosceles* venoms, due to their biochemical and biological properties, are examples of great biotechnological potential for pharmaceutical and chemical industries, among others. Biochemical properties modulating cell phospholipids, are extremely interesting and useful properties in technological applications, especially now with the availability of high concentrations of soluble and functional recombinant isoforms of several *Loxosceles* PLDs. These enzymes can be used not only as biological reagents for the experimental generation of these lipids, but also for studies related to the receptors and cellular cascades involved in these activities. In this field, the activity of these PLDs generating the production of cyclic forms of ceramide-1-phosphate and lysophosphatidic acid [67], enables the use of these enzymes as catalysts in reactions of transphosphatidylation for the industrial production of cyclic forms of lipids.

The insecticidal activity of Brown spider PLDs, described for the recombinant enzymes of L. arizonica and L. intermedia [12], This work, Figure 1E, is another potential applicability of these molecules that can be exploited. After the full mechanism of cytotoxicity and neurotoxicity is known, it will be possible to optimize these molecules and use them in this field that is constantly demanding new technologies.

Additionally, the new enzyme engineering methodologies which permit modifications in the primary structures of these PLDs, and consequent alterations in the biochemical and biological properties, can alter properties causing inhibition or even activation of mutated isoforms, thus creating mutants with enormous biotechnological potential. Finally, complementing these data, the modern biophysical techniques of studies of protein structures can provide information that was previously unavailable, opening great possibilities for technological applicability of these molecules. 

As already discussed here, it is a great possibility to apply these toxins to produce a second generation anti-*Loxosceles* serum, protective vaccines against the toxic effects of envenomation and laboratory methods for immunodiagnostic of loxoscelism. In both cases, either by engineering enzymes originating mutated recombinant forms with preserved conformational structures, or using synthetic chimeras, the amount of antigen generated is large, favoring the production of sera with higher titers of anti-PLD antibodies and vaccines. 

A new possibility of biotechnological application of loxoscelic PLDs is their use as biological reagents for research protocols in the field of biological sciences, especially in cell biology, pharmacology, immunology, hematology, and biochemistry. These enzymes have modulatory activities on various cells, such as erythrocytes, neutrophils, platelets, endothelium, fibroblasts, lymphocytes and renal cells, among others. PLDs as bio-tools could be used in research protocols that require positive controls of cell changes. The direct or complement-dependent hemolytic activities of these toxins [29,61,82,138], provide the use of PLDs in functional studies on red blood cells. The effects of inflammatory response and indirect activation of neutrophils [13,14,28,48,81] enable these enzymes to function as excellent tools for studies on the release of inflammatory mediators such as cytokines and chemokines. Moreover, the platelet-aggregating activities of PLDs from *Loxosceles* [13,48,53] indicate that these toxins could be used as modulating agents for platelet aggregation in experimental or diagnostic protocols in the hematology area. The action of PLDs of *Loxosceles* venoms on blood vessel endothelial cells [28], enable these PLDs as useful biological tools in experimental protocols that study endothelial cells. In addition, the action of recombinant isoforms of PLDs on cultured fibroblasts [80,81], which triggers the production and secretion of cytokines and chemokines, render these toxins as useful tools for experimental protocols to study fibroblasts. Finally, the effects of PLDs on renal cells [47,59] qualify these molecules as potentially useful in experimental studies on nephrotoxicity. Not to mention the hypothesis of tumor cell modulating activity after treatment with PLDs of Brown spider venoms, which by stimulating the formation of bioactive lipids, as lysophospholipids or sphingophospholipids derivatives, can modulate tumor cell growth, migration or survival [139]. In fact, several phospholipases-D have been described as modulating malignant characteristics of tumor cells [140]. An important example is autotaxin, a PLD secreted by tumor cells, which seems to confer greater migratory potential for tumor cells, and thus increase the potential for metastasis occurrence [141]. In summary, the cytomodulatory potential of *Loxosceles* PLDs on different cells enables these enzymes as excellent tools in cell biology.

## 12. Actions Expected for the Future of *Loxosceles* PLD Researches

**Figure 4:**
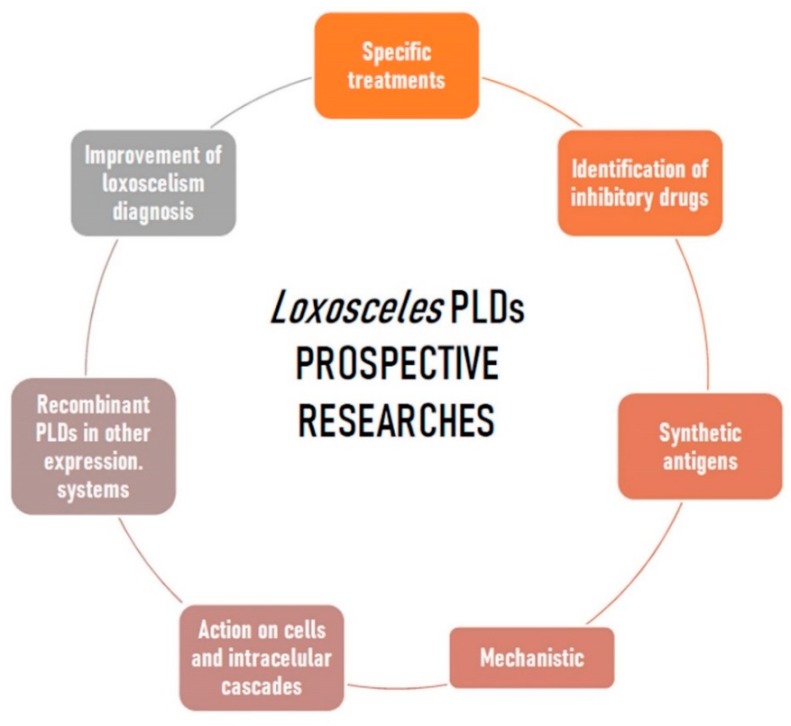
Actions expected for the future of *Loxosceles* PLD researches.

The *Loxosceles* genus spiders have a worldwide distribution, with 156 different species of these arachnids described in the world [142]. Loxoscelism is a global problem, but it prevails in the areas with warmer climates of South America, North America, Mediterranean Europe and Africa. In some regions of countries such as Brazil, Chile, and Peru, loxoscelism is considered a public health problem due to the high frequency of accidents [5,85]. Although there are a large number of different species of Brown spiders, the species with medical relevance are few in number, prevailing in North America *Loxosceles reclusa*, *L. deserta,* and *L. arizonica*, and in South America *L. intermedia*, *L. gaucho,* and *L. laeta* [5,6,85,143,144]. The PLDs present in *Loxosceles* spider venoms are the major toxins concerning the effects triggered by envenomation. Despite the breakthrough in the last 40 years there are many data and expectations concerning the research of these enzymes which need to be addressed for the future. Undoubtedly, the improvement of specific treatments for loxoscelism is a necessity and it clearly involves the PLDs. A targeted therapy may be achieved by using these toxins for synthetic chemical antagonist designs or as recombinant antigens, which may enhance the quality and efficiency of anti-loxoscelic sera and allow the production of vaccines. Moreover, available information on the structural characteristics of the *Loxosceles* venoms PLDs can be used to search chemical libraries and identify drugs with inhibitory action on these toxins, therefore, there is a great potential for discoveries of new treatments. In this regard, synthetic antigens containing linear sequences of the PLDs may also be useful in this approach. For these purposes, analyzes of the structures and functions, bioinformatics and molecular dynamics relations can give an exact description of the catalysis mechanisms, which amino acid residues participate in the degradation of the substrates, as well as details on the PLD-substrate interactions. It is also expected that all the scientific knowledge on the PLDs will be used to improve the diagnosis of loxoscelism and, in the end, a laboratorial method could be established, ideally non-invasive and sensitive, which is able to diagnose the accidents. There is also a lot yet to be studied and discovered on the degradation of cellular phospholipid substrates, cellular receptors and cascades of activated intracellular signaling, bringing more rational parameters on PLD toxins and on the loxoscelism. In the basic research field, new isoforms of recombinant PLDs can be obtained not only in bacterial models as available today, but using other heterologous expression systems, such as yeast and insect cells, and thus yield information not currently available. Standardizations of co-crystals of PLDs and phospholipid substrates, as well as drugs that could interact with these toxins, will provide information on the mechanistic of these enzymes. These perspectives discussed here are summarized in Figure 4. In short, although much scientific knowledge has been described concerning the PLDs from *Loxosceles* spider venoms, bringing a huge gain in learning about these molecules and on loxoscelism, much is yet to be done to alleviate the suffering of injured patients. We hope that in the coming years gaps will be closed and science will improve learning in this amazing field of toxinology. Brown spider PLDs: much learning, but still much to be done on this great avenue.

## Figures and Tables

**Table 1:** Recombinant *Loxosceles* phospholipases-D (PLDs) and their main activities.

Recombinant *Loxosceles* PLDs			Known Activity											References
Species	Name	GenBank Acession Number	*Dermonecrosis*	*Hemolysis*	*Sphingo-myelinase*	*In vitro Platelet Aggregation*	*Vasc. Perm. Increase*	*Inflammat.*	*Lethality*	*Nephrotox*	*Edema*	*Cardiotox*	*Insecticidal*	
***L. intermedia***	LiRecDT1; LiSicTox-alphaIA1a	ABA62021, P0CE80	Yes	Yes (Direct/C-dep)	Yes	Yes	Yes	Yes	Yes	Yes	Yes	−	Yes	[9,47,53,54]^,^ This work
	LiD1	AAQ16123	Yes	Yes (C-dep)	Yes	Yes	−	−	−	−	Yes	Yes	−	[45,55,56,57]
	LiRecDT2; LiSicTox-alphaIA2aii	ABB69098, P0CE83	Yes	Yes (Direct)	Yes	Yes	Yes	Yes	Yes	−	Yes	−	−	[13,15,54]
	LiRecDT3	ABB71184	No	No	No	No	No	±	No	−	No	−	−	[13,15]
	LiRecDT4	ABD91846	Yes	−	Yes	±	Yes	Yes	−	−	Yes	−	−	[14]
	LiRecDT5	ABD91847	No	−	No	No	No	±	−	−	Yes	−	−	[14]
	LiRecDT6	ABO87656	Yes	−	Yes	Yes	Yes	Yes	Yes	−	Yes	−	−	[48]
	LiRecDT7	AGN52903	Yes	Yes (Direct)	Yes	−	Yes	Yes	−	−		−	−	[16]
***L. laeta***	Smase I/ LlSicTox-alphaIII1i	AAM21154	Yes	Yes (C-dep)	Yes	−	−	Yes	−	−	Yes	−	−	[18,46]
	Ll1/LlSicTox-alphaIII1ii	ABD15447	−	−	Yes	−	−	−	Yes	−	−	−	−	[51]
	LlPLD1	ADP00408	−	Yes (C-dep)	Yes	−	−	Yes	−	−	−	−	−	[52,58]
	LlPLD2	ADP00409	−	No	No	−	−	Yes	−	−	−	−	−	[52,58]
	Ll2/LlSicTox-alphaIII2	ABD15448	−	−	Yes	−	−	−	Yes	−	−	−	−	[51]
	Smase II/ LlSicTox-betaIA1	AAM21156	±	Yes (C-dep)	Yes	−	−	Yes	−	−	Yes	−	−	[18,46]
***L. reclusa***	Smase D	AAW56831	−	Yes (C-dep)	Yes	−	−	−	−	−	−	−	−	[27]
	Lr1	AAT66075	−	−	Yes	−	−	−	Yes	−	−	−	−	[51]
***L. boneti***	Lb1	AAT66073	−	−	Yes	−	−	−	Yes	−	−	−	−	[51]
	Lb3	AAT66074	−	−	No	−	−	−	−	−	−	−	−	[51]
***L. arizonica***	LarSicTox-alphaIB2bi	Q4ZFU2	−	−	Yes	−	−	−	−	−	−	−	Yes	[12]
	LarSicTox-betaID1	AJV88487	−	−	Yes	−	−	−	−	−	−	−	−	[30]
***L. gaucho***	LgRec1	AFY98967	Yes	Yes (Direct)	Yes	Yes	−	−	−	−	Yes	−	−	[53]
